# Pregnancy Complicated by Citrin Deficiency and Gestational Diabetes Mellitus: Perioperative Metabolic Management and Postoperative Analgesic Challenges Following Caesarean Delivery

**DOI:** 10.7759/cureus.117730

**Published:** 2026-10-10

**Authors:** Ei Ei Phyo Naing, Ei Mon Mon Kyaw

**Affiliations:** 1 Obstetrics and Gynaecology, Luton and Dunstable University Hospital, Luton, GBR

**Keywords:** caesarean section, citrin deficiency, citrullinaemia type ii, gestational diabetes mellitus, inherited metabolic disease, pregnancy, slc25a13

## Abstract

Citrin deficiency is a rare autosomal recessive metabolic disorder caused by pathogenic variants in the SLC25A13 gene. Pregnancy in affected women is uncommon and presents unique challenges because conventional obstetric management may conflict with metabolic requirements. The coexistence of gestational diabetes mellitus (GDM) introduces additional complexity.

A 25-year-old, gravida 2 para 0+1 woman with genetically confirmed citrin deficiency (homozygous *SLC25A13* c.1763G>A mutation) was managed jointly by obstetric and inherited metabolic disease (IMD) teams. Her pregnancy was complicated by severe nausea and vomiting during the first trimester and subsequent GDM diagnosed using capillary glucose monitoring rather than an oral glucose tolerance test. Representative fasting glucose values were 6.3-6.8 mmol/L, with postprandial values up to 10.7 mmol/L. Specialist dietary modification and insulin therapy were instituted following multidisciplinary review. A personalised metabolic plan emphasised avoidance of prolonged fasting, restriction of excessive carbohydrate intake, maintenance of adequate energy intake, and avoidance of routine glucose-containing intravenous fluids.

At 38+5 weeks’ gestation, she underwent emergency caesarean section for failure to progress in labour under spinal anaesthesia. A healthy infant weighing 3074 g was delivered with Apgar scores of 9, 10 and 10 at 1, 5 and 10 minutes, respectively. Postoperative pain management was challenging because the specialist metabolic plan advised caution with paracetamol. Following inadequate analgesia with ibuprofen and dihydrocodeine, paracetamol was cautiously introduced at reduced doses and subsequently increased to 500 mg four times daily, with additional opioid analgesia as required. Daily plasma ammonia and liver function monitoring was undertaken during the five-day postoperative admission; ammonia values remained within the laboratory reference range, with no hyperammonaemia (range <10-32 µmol/L) or significant hepatic biochemical deterioration.

This case highlights the complexity of managing pregnancy in women with citrin deficiency, particularly when complicated by GDM. It also demonstrates a practical approach to postoperative analgesia following caesarean delivery using cautious dose escalation and biochemical surveillance.

## Introduction

Citrin deficiency is a rare autosomal recessive metabolic disorder caused by pathogenic variants in the SLC25A13 gene, which encodes citrin, a mitochondrial aspartate-glutamate carrier involved in the malate-aspartate shuttle and hepatic urea-cycle function [1,2]. Impaired citrin activity disrupts the transport of aspartate from the mitochondrial matrix to the cytosol and reduces the transfer of cytosolic reducing equivalents into mitochondria via the malate-aspartate shuttle. This alters the cytosolic NADH/NAD⁺ balance, impairing hepatic carbohydrate metabolism and limiting the availability of cytosolic aspartate required for argininosuccinate synthesis in the urea cycle.

Consequently, excessive carbohydrate or glucose loading may exacerbate metabolic dysfunction, while impaired urea-cycle activity increases susceptibility to hyperammonaemia. Patients with citrin deficiency characteristically prefer protein- and fat-rich foods and may exhibit an aversion to carbohydrate-rich diets [3-6]. These metabolic characteristics have important implications during pregnancy, when adequate energy intake must be maintained while avoiding prolonged fasting, catabolic stress and excessive carbohydrate exposure. The coexistence of gestational diabetes mellitus (GDM) further complicates management because standard treatment pathways may conflict with the metabolic requirements of citrin deficiency. 

Published evidence concerning pregnancy in women with citrin deficiency remains sparse. A previous case report described pregnancy-associated hyperammonaemic encephalopathy in a woman with type II citrullinaemia, followed by subsequent successful pregnancies [7]. Although this report provides valuable information about pregnancy outcomes and metabolic risks, detailed evidence regarding emergency caesarean delivery, perioperative metabolic planning and postoperative analgesic selection in this population remains insufficient. In particular, the use of paracetamol and opioid analgesia following caesarean delivery has not been adequately characterised. The present case therefore contributes additional clinical detail regarding the multidisciplinary management of these overlapping challenges.

We report a pregnancy complicated by genetically confirmed citrin deficiency and insulin-treated GDM, highlighting three key clinical challenges: maintaining metabolic stability during pregnancy while minimising fasting and catabolic stress; adapting GDM management to the specific nutritional requirements of citrin deficiency; and individualising perioperative and postoperative care, particularly analgesia following caesarean delivery. This case illustrates the importance of multidisciplinary collaboration in addressing these challenges in the context of limited pregnancy-specific evidence.

## Case presentation

A 25-year-old woman, gravida 2 para 0+1, with genetically confirmed citrin deficiency was referred for specialist antenatal care. Molecular analysis had previously identified a homozygous pathogenic SLC25A13 c.1763G>A mutation. Her obstetric history included one previous spontaneous miscarriage in the early trimester, which was managed conservatively without surgical intervention. There were no previous live births or reported metabolic complications, including episodes of metabolic decompensation, during the previous pregnancy.

The patient had genetically confirmed citrin deficiency associated with a homozygous SLC25A13 c.1763G>A variant, identified through family screening. Detailed information regarding her age at diagnosis, pre-pregnancy clinical manifestations, baseline dietary pattern and previous metabolic treatment was unavailable. The available records were insufficient to establish whether she had previously experienced hyperammonaemia, hepatic dysfunction, pancreatitis, dyslipidaemia or metabolic crises. Consequently, her clinical phenotype could not be definitively classified as symptomatic adult-onset type II citrullinaemia (CTLN2). Antenatal and peripartum management was therefore guided by her confirmed genetic diagnosis and the individualised recommendations of the specialist inherited metabolic disease team.

At booking, her weight was 72 kg and body mass index (BMI) was 28.8 kg/m². Routine antenatal investigations, including HbA1c, were normal.

Antenatal course

During the first trimester of pregnancy, at approximately 13 weeks’ gestation, the patient experienced severe nausea and vomiting requiring hospital admission. Management included antiemetic therapy with cyclizine, ondansetron, prochlorperazine and doxylamine/pyridoxine, alongside nutritional support with oral supplements. In the context of citrin deficiency, reduced oral intake raised concerns regarding potential catabolic stress and metabolic instability. However, contemporaneous data regarding weight loss, ketones, electrolytes, plasma ammonia and liver enzymes were unavailable for detailed assessment. Therefore, metabolic decompensation during this episode could not be confirmed.

Gestational diabetes mellitus (GDM) was identified during antenatal glucose surveillance. In view of the potential metabolic consequences of a concentrated oral glucose load in citrin deficiency, a standard oral glucose tolerance test was not performed. Instead, capillary blood glucose monitoring was used to assess glycaemic status. Recorded fasting glucose values included 6.3-6.8 mmol/L, with postprandial readings reaching 10.7 mmol/L. Given the limited published evidence regarding metformin use in patients with citrin deficiency, its safety in this context was uncertain. Following multidisciplinary discussion involving the obstetric, diabetes and inherited metabolic disease teams, specialist dietary modification and insulin therapy were instituted. Subsequent documented readings included a fasting glucose concentration of 5.8 mmol/L and postprandial values of 6.7-8.0 mmol/L, indicating improvement in some measurements, although not consistently meeting conventional pregnancy glycaemic targets. Detailed longitudinal glucose records, insulin titration data and a later-pregnancy HbA1c were not available for assessment.

An individualised metabolic and nutritional management plan was developed in collaboration with the inherited metabolic disease (IMD) team and specialist dietitians. Recommended dietary targets included protein contributing approximately 15-20% of total energy intake, fat 40-50%, and carbohydrates 30-40%. The emphasis was on avoiding excessive carbohydrate loads while maintaining adequate caloric intake through regular meals and snacks, thereby minimising prolonged fasting and catabolic stress. Following the diagnosis of GDM, dietary counselling was reinforced to balance glycaemic control with the specific nutritional requirements of citrin deficiency. The metabolic plan also advised against routine glucose-containing intravenous infusions and included an individualised emergency hypoglycaemia management strategy. Detailed records of actual daily caloric intake, fasting duration and ketone measurements were unavailable.

Delivery

At 38+5 weeks’ gestation, the patient underwent an emergency lower-segment caesarean section for failure to progress in labour. The procedure was performed under spinal anaesthesia, with an estimated blood loss of 500 mL and no reported intraoperative complications. Perioperative management was guided by the individualised metabolic protocol, which recommended minimising prolonged fasting, avoiding routine glucose-containing intravenous fluids and ensuring appropriate management of hypoglycaemia. However, detailed records of fasting duration, intravenous fluid administration and perioperative glucose and insulin management were unavailable. Plasma ammonia concentrations were 19 µmol/L on the day of delivery and 25 µmol/L on postoperative day one. A healthy infant weighing 3,074 g was delivered with Apgar scores of 9, 10 and 10 at 1, 5 and 10 minutes, respectively. No neonatal complications were identified.

Postoperative course

The most significant management challenge occurred in the postoperative period. The pre-existing metabolic management plan recommended avoidance of paracetamol because of concerns regarding hepatic metabolism and the absence of evidence supporting its use in citrin deficiency. Initial analgesia with regular dihydrocodeine and ibuprofen proved inadequate.

Following a multidisciplinary discussion with the IMD team, low-dose paracetamol (250 mg four times daily) was cautiously introduced, alternating with sugar-free morphine 2.5 mg three times daily.

Pain control remained suboptimal, and the regimen was subsequently escalated to paracetamol 500 mg four times daily and sugar-free morphine 5 mg three times daily. A sugar-free oral morphine preparation was selected as part of the individualised analgesic strategy, reflecting the broader dietary approach of limiting excessive carbohydrate exposure in citrin deficiency. However, the specific carbohydrate excipients and their quantities in alternative preparations were not established. Consequently, the clinical significance of selecting a sugar-free formulation in this case remains uncertain.

Serial plasma ammonia concentrations were monitored during the peripartum period and remained below the laboratory upper reference limit (Table 1). Alanine aminotransferase concentrations ranged from 17 to 27 U/L, with no documented biochemical evidence of hyperammonaemia during this period.

**Table 1:** Serial plasma ammonia concentrations during the peripartum period

Clinical period	Plasma ammonia (µmol/L)
Antepartum day 1	<10
Antepartum day 2	21
Day of caesarean delivery	19
Postoperative day 1	25
Postoperative day 2	22
Postoperative day 3	25
Postoperative day 4	25
Postoperative day 5	32

Although the initial management plan anticipated a three-day postnatal admission, the patient remained hospitalised for five days to facilitate optimisation of analgesia and continued biochemical surveillance. She was subsequently discharged home well with her infant. The key clinical events from booking through the postoperative period are summarised in Table 2.

**Table 2:** Timeline of clinical events

Gestational age/timepoint	Clinical event	Metabolic findings and management	Outcome
Booking visit	Initial antenatal assessment	Weight 72 kg; BMI 28.8 kg/m²; HbA1c 36 mmol/mol	Baseline assessment
Approximately 13 weeks	Severe nausea and vomiting requiring hospital admission	Antiemetic therapy and oral nutritional supplementation; contemporaneous biochemical data unavailable	Metabolic decompensation not confirmed
Antenatal period	GDM identified through capillary glucose monitoring	Fasting glucose 6.3–6.8 mmol/L; postprandial glucose up to 10.7 mmol/L	Specialist dietary modification and insulin therapy
Antenatal period	Individualised metabolic management plan	Recommended protein 15–20%, fat 40–50%, carbohydrates 30–40% of total energy intake; precautions against prolonged fasting	Multidisciplinary management
38+5 weeks	Emergency lower-segment caesarean section for failure to progress	Perioperative metabolic precautions recommended; plasma ammonia 19 µmol/L	Estimated blood loss 500 mL; infant 3,074 g; Apgar scores 9, 10 and 10
Postoperative day 1	Inadequate pain control with initial analgesia	Individualised analgesic adjustment; plasma ammonia 25 µmol/L	Further analgesic management required
Postoperative days 2–5	Continued postoperative care and biochemical surveillance	Plasma ammonia 22–32 µmol/L; no documented hyperammonaemia	Biochemical stability during documented period

## Discussion

This case highlights several important challenges encountered during pregnancy in a woman with citrin deficiency.

First, severe nausea and vomiting during early pregnancy created a risk of reduced caloric intake and catabolic stress. In patients with citrin deficiency, fasting and catabolism may increase the risk of metabolic instability, emphasising the importance of specialist dietetic and metabolic input [3-5].

Second, the diagnosis of GDM introduced competing metabolic priorities. Standard management of GDM involves dietary modification and may include metformin or insulin therapy [8]. However, patients with citrin deficiency typically require restriction of excessive carbohydrate intake, and evidence regarding the safety of metformin in this population remains limited [3,4]. This uncertainty does not establish metformin as contraindicated in citrin deficiency. Following multidisciplinary discussion, insulin was selected for glycaemic management as part of an individualised treatment approach.

Perioperative planning also required modification of standard obstetric practice. The individualised metabolic management plan recommended minimising prolonged fasting and avoiding routine glucose-containing intravenous fluids because of concerns regarding excessive carbohydrate exposure in citrin deficiency. However, this did not constitute an absolute contraindication to intravenous glucose, which would remain necessary if clinically indicated, particularly in the management of hypoglycaemia. Careful assessment of glucose levels and nutritional status is therefore essential to balance the risks of excessive carbohydrate administration against hypoglycaemia and catabolic stress. Close collaboration between obstetricians, anaesthetists and metabolic specialists was important in planning perioperative care. However, incomplete documentation of actual intravenous fluid administration and perioperative glucose monitoring limits assessment of the implementation of this strategy.

Postoperative analgesia presented a particular challenge because evidence guiding the selection and dosing of commonly used analgesics in patients with citrin deficiency is limited. Although paracetamol is routinely included in multimodal analgesic regimens following caesarean delivery, the patient's specialist metabolic management plan advised caution regarding its use. While concerns had been raised about potential metabolic decompensation, there is insufficient evidence to establish altered paracetamol metabolism or a specific contraindication in patients with SLC25A13 deficiency. The initial restriction therefore represented an individualised precautionary decision rather than an established pharmacological contraindication.

Initial postoperative analgesia with dihydrocodeine and ibuprofen provided inadequate pain relief. Following consultation with the inherited metabolic disease (IMD) team, paracetamol and morphine were introduced cautiously as part of a multimodal analgesic strategy, accompanied by biochemical monitoring. Serial plasma ammonia concentrations and liver function tests were monitored during the postoperative period, with no documented hyperammonaemia. However, these biochemical parameters are not validated markers of analgesic safety in citrin deficiency, and their use in this case reflected an individualised monitoring approach rather than an established protocol. The monitoring approach was informed in part by published guidance for inherited metabolic and urea cycle disorders [6], although such recommendations cannot be assumed to apply directly to citrin deficiency. The postoperative analgesic regimen is summarised in Table 3.

**Table 3:** Postoperative analgesic escalation following caesarean delivery

Analgesic Stage	Regimen	Clinical Outcome	Biochemical Monitoring
Initial management	Dihydrocodeine and ibuprofen	Inadequate pain relief	Daily ammonia and liver function monitoring
First escalation	Paracetamol 250 mg four times daily + sugar-free morphine 2.5 mg three times daily	Partial improvement	Normal ammonia and liver function tests
Second escalation	Paracetamol 500 mg four times daily + sugar-free morphine 5 mg three times daily	Adequate pain control achieved	Normal ammonia and liver function tests
Outcome	Continued clinical recovery	No evidence of metabolic decompensation	Normal biochemical surveillance

Alternative postoperative analgesic strategies include neuraxial opioids and regional anaesthetic techniques, which may reduce systemic opioid requirements following caesarean delivery. However, evidence regarding their use specifically in patients with citrin deficiency is limited. The analgesic approach adopted in this case reflected individualised specialist advice and the need to address inadequate pain relief, rather than evidence of superiority over alternative techniques.

This experience suggests that careful escalation of analgesic therapy may be feasible in selected patients with citrin deficiency when supported by close multidisciplinary oversight and biochemical surveillance. Inadequate pain control itself may contribute to physiological stress and catabolism; therefore, balancing effective analgesia against theoretical metabolic risks is an important clinical consideration.

Literature review

Published evidence regarding pregnancy in women with citrin deficiency remains limited. A previous case report described pregnancy-associated hyperammonaemic encephalopathy in a woman with type II citrullinaemia, highlighting the potential for metabolic instability during pregnancy [9]. However, detailed evidence concerning perioperative metabolic management and postoperative analgesia following caesarean delivery remains insufficient.

In the present case, paracetamol and morphine were introduced cautiously following specialist consultation, without documented postoperative hyperammonaemia or deterioration in liver function. Nevertheless, tolerance in this individual patient cannot establish the general safety of these medications in citrin deficiency. Further reports are needed to inform clinical management.

## Conclusions

Pregnancy complicated by citrin deficiency requires individualised multidisciplinary care. The coexistence of GDM introduces additional management complexity because conventional diabetic treatment strategies may conflict with metabolic requirements.

This case highlights the challenges of managing pregnancy complicated by citrin deficiency, particularly in relation to nutritional requirements, gestational diabetes mellitus and perioperative care. Individualised metabolic planning and multidisciplinary collaboration were central to clinical decision-making. Paracetamol and morphine were administered cautiously under specialist supervision without documented biochemical deterioration during the monitoring period. However, this observation in a single patient does not establish the general safety of these medications in citrin deficiency. Further evidence is needed to inform management strategies for pregnant patients with this rare metabolic disorder.
